# Predominant Role of Immunoglobulin G in the Pathogenesis of Splenomegaly in Murine Lupus

**DOI:** 10.3389/fimmu.2019.03020

**Published:** 2020-01-24

**Authors:** Qian Zhang, Liping Xiang, Muhammad Haidar Zaman, Wenhui Dong, Guodan He, Guo-Min Deng

**Affiliations:** ^1^Key Laboratory of Antibody Techniques, National Health Commission, Nanjing Medical University, Nanjing, China; ^2^Department of Clinical Laboratory, Nanjing Jiangning Hospital, The Affiliated Jiangning Hospital of Nanjing Medical University, Nanjing, China; ^3^Department of Rheumatology, Union Hospital Affiliated to Tongji Medical College, Huazhong University of Science and Technology, Wuhan, China

**Keywords:** immunoglobulin G, systemic lupus erythematosus, spleen, inflammation, macrophages, germinal center

## Abstract

Systemic lupus erythematosus (SLE) is characterized by high levels of autoantibodies and multiorgan tissue damage. The pathogenesis of splenomegaly in SLE remains unknown. In this study, the role of immunoglobulin G (IgG) generation and deposition in the inflammation of the spleen and associated dysfunction in SLE was investigated. In the lupus mice, we observed the development of spontaneous splenomegaly, and we found that lupus serum IgG is an important pathological factor involved in the initiation of inflammation and further germinal center (GC) and plasma cell formation. We discovered that macrophages of the splenic marginal zone are dispensable for the GC response induced by lupus IgG, but red pulp macrophages are important for GC responses. Furthermore, we found that pathogenic lupus IgG promotes inflammation and GC formation through the macrophage-mediated secretion of TNF-α. Syk inhibitor treatment suppressed the changes in the histopathology of the spleen induced by lupus IgG. This study will contribute to the understanding of the pathogenesis of splenomegaly in lupus and promote the development of an effective therapeutic strategy for SLE.

## Introduction

Systemic lupus erythematosus (SLE) is a chronic autoimmune disease characterized by high levels of autoantibodies and multiorgan damage. The unknown interaction of genetics factors, environmental elements, and hormone levels results in abnormal immune cell activation and the release of cytokines, leading to damage multiple organs, such as the kidney, skin, lungs, brain, and joints (1, 2). The immunologic disturbances in SLE involve autoantibodies and the formation and deposition of immune complexes. SLE patients and murine lupus models exhibit increased autoantibody and nuclear self-antigens levels in the circulation, which causes tissue damage, immune complex deposition, complement activation, and cytokine secretion (3, 4). It has been reported that circulating immune complexes can activate plasmacytoid dendritic cells to induce type I IFN responses, which stimulate the differentiation of monocyte-derived dendritic cells and promote B cell activation and humoral autoimmunity (5). Both innate and adaptive immune systems are involved in the immune response in SLE pathogenesis.

The spleen is an important peripheral lymphoid organ for antibacterial and antifungal immunity. As the site of immunocyte proliferation and differentiation, the spleen combines the innate and adaptive immune responses in an exclusively organized manner. The red pulp macrophage and marginal zone macrophage (MZM) populations in the spleen enable the efficient removal of aging erythrocytes and blood-borne pathogens, respectively (6, 7). MZMs located in the marginal zone barrier, express the type I scavenger receptor MARCO and type C lectin SIGN-R1, which recognizes pathogens (8). White pulp containing T, B, and follicular dendritic cells are involved in adaptive immunity (9, 10). After antigen-specific differentiation in the splenic follicles, plasma cells produce and migrate into the red pulp. Autoantibodies are produced by B cells recruited to the germinal center (GC). The GC is an important site of B cell differentiation into long-lived memory and plasma cells. Traditionally, high-affinity autoantibodies are associated with the GC response, but recent studies have indicated that B cell activation and differentiation also occurs in the extrafollicular pathway in SLE. In SLE, autoantibodies are secreted by plasma cells in the spleen through both extrafollicular and GC pathways (11).

Splenomegaly is infrequently observed in SLE patients and lupus mice, although it has been reported as a manifestation of active SLE (12, 13). Splenomegaly may be caused by increased splenic function, congestion, or infiltration (14–16). Although the spleen is not considered a common target organ in SLE, but the function of the spleen in producing antibodies cannot be neglected. Thus, it is important to understand the pathogenesis and features of splenomegaly and inflammation of spleen in lupus.

T cells, B cells, and macrophages are known to contribute in the pathogenesis of SLE. Autoantibodies are involved in the pathogenesis of autoimmune diseases (17). Previous studies showed that lupus patient serum induces tissue damage (18–20). Serum IgG from lupus patients activates inflammatory cells, such as monocytes/macrophages, dendritic cells and neutrophils, which produce cytokines and subsequently induce tissue damage (21–23). Fcγ receptors (FcγRs) are receptors for IgG, and the balance between activating and inhibitory FcγRs determines the threshold of immune cells activation (24). B cells express only the inhibitory receptor FcγRIIb. It has been reported that FcγRIIB-deficient mice display an increased frequency of autoreactive B cells and lupus-like manifestations (25, 26). Although monocyte/macrophage abnormalities play a pivotal role in the pathogenesis of SLE, the role of macrophages in spleen inflammation and splenomegaly is still unknown. Spleen tyrosine kinase (Syk) is a member of the Src family of non-receptor tyrosine kinases that associate with surface receptors, including the B cells receptor (BCR) and Fcγ receptors, and is involved in the signal transduction pathways of PI3K/Akt, Ras/ERK, PLCc/NFAT, and IKK/NF-κB (27, 28). Treatment with the Syk inhibitor R788 completely abrogates skin inflammation induced by serum from lupus patients and suppresses established skin injury in lupus-prone mice (29), but whether inhibiting Syk suppresses spleen inflammation remains unknown.

In the present study, we investigated the histopathological features and pathogenesis of splenomegaly in lupus mice. We found that in lupus mice, IgG is an important pathological factor in red pulp inflammation and GC responses; pathogenic lupus IgG promoted GC formation through the macrophage-mediated secretion of TNF-α. Inhibiting Syk suppressed inflammation of the spleen induced by lupus IgG. This study broadens the understanding of the pathogenesis of splenomegaly in lupus mice.

## Materials and Methods

### Mice and Reagents

C57BL/6 (B6) mice were obtained from the Animal Center of Nanjing Medical University. B6.MRL-Fas^lpr^/J (B6.MRL/lpr; 000482) mice were purchased from the Model Animal Research Center of Nanjing University. MRL/MpJ (MRL; 000486), MRL/MpJ-Fas^lpr^/J (MRL/lpr; 000485), FcγRIIb^−/−^ (002848), FcγRIII^−/−^ (003171), and TNF-α^−/−^ (005540) mice were purchased from Jackson Laboratories (USA). Pathogen-free environments were provided at the Animal Core Facility of Nanjing Medical University. All animal experiments were approved by the Nanjing Medical University Institutional Animal Care and Use Committee (IACUC-1710012). All experiments used in this study were approved by the Institutional Ethics Committee of Nanjing Medical University.

SLE patient and healthy human sera were provided by the First Affiliated Hospital of Nanjing Medical University and the Third Affiliated Hospital of Nanjing Medical University. From the provided serum, SLE patients with ≥4 of the 11 revised criteria of the American College of Rheumatology (ACR) for the classification of SLE were selected. All patients had SLE disease-activity index scores ranging from 0 to 20. Serum from healthy individuals was used as a control. Informed consent was received from all patients under the Nanjing Medical University Review Board-approved protocol. Mice sera were collected from B6.MRL/lpr and C57BL/6 mice.

Lupus IgG was extracted from the SLE patient serum using Protein G Agarose beads (Millipore, USA) following the manufacturer's protocol, as described previously. The Syk inhibitor R406 (sc-364595A) was purchased from Santa Cruz Biotechnology (Dallas, USA). Clodronate liposomes (CLs) were purchased from FormuMax (California, USA).

### Histopathology, Histochemistry, Immunohistochemistry (IHC), and Microscopy

For histological examinations, spleens were fixed in 4% paraformaldehyde. After fixation, the samples were dehydrated in ethanol, embedded in paraffin, cut into 5-μm sections, and stained with hematoxylin and eosin (HE). The spleen inflammation severity was scored from 0 to 4: grade 0 = normal, grade 1 = mild inflammation in the red pulp, grades 2–3 = different quantities of infiltrating inflammatory cells in the red pulp and under the capsule, and grade 4 = increased numbers of infiltrating inflammatory cells in the red pulp and a large number of megakaryocytes (megakaryocyte hyperplasia). For the IHC assay, the tissue slides were incubated with primary anti-mouse IgG (ab190475, Abcam), anti-MARCO (sc-65353, Santa Cruz), anti-CD138 (10593-1-AP, Proteintech), anti-F4/80 (ab6640, Abcam), and anti-human IgG (ab109489, Abcam) antibodies followed by incubation with biotinylated secondary antibodies and avidin–biotin–peroxidase complex treatment. Then, 3,3′-diaminobenzidine was used for development, and all slides were counterstained with Mayer's hematoxylin. Biotin-conjugated PNA (Vector Laboratories B-1075) was used to identify the GC, which was captured with a Zeiss LSM700 microscope (Carl Zeiss, USA).

Histochemistry of reticular fibers in paraffin sections (8 μm) was assessed by the silver impregnation method previously described by Gordon and Sweets (30). Frozen sections were air-dried and fixed with ice-cold acetone and then detected by acid phosphatase using the previously described Gomori acid phosphatase method (31).

### Injection Protocol and Macrophage Depletion

For ink injection, B6 mice and B6.MRL/lpr mice (30 weeks) were injected with Indian ink (0.1 ml/10 g) containing 50–100 nm carbon particles. India ink diluted 1:10 in PBS was injected intravenously after anesthesia with 10% chloral hydrate (Sigma, Aldrich). The spleens were collected 30 min after injection, and formalin-fixed paraffin sections (5 μm) were assessed using light microscopy and HE staining.

Intrasplenic injections were administered to C57BL/6 mice to induce IgG deposition and inflammation in the spleen after mice anesthetized intraperitoneally with 10% chloral hydrate (Sigma, Aldrich). Following disinfection, an ~1 cm incision (without opening the peritoneal cavity) was made at the left loin, and then lupus serum was injected into the spleen *in situ*. Mice received the same volume of PBS or healthy human serum as the control. C57BL/6 mice used in this study were female with 6–8 weeks of age, unless otherwise indicated.

Macrophages were depleted with 1 mg (40 mg/kg) CLs administered to C57BL/6 mice by intraperitoneal injection 48 h before lupus IgG (200 μg) injection. To selectively deplete the MZMs, 162 μg (6.5 mg/kg) of CLs was injected intraperitoneally. The same volume of empty liposome-phosphate-buffered saline (control liposomes) was injected as a control.

### Flow Cytometry

The spleen was isolated and mashed into RPMI 1640 medium containing 2% FBS. After erythrocytes were lysed, a single cell suspension of splenocytes was prepared and then stained with the cell surface markers. For GC B cell staining, cells were stained with anti-B220-PE (RA3-6B2, Biolegend), anti-Fas-APC (15A7, eBioscience), and anti-GL-7-FITC (GL-7, Biolegend). For marginal zone B (MZB) cell staining, cells were stained with anti-B220-FITC (RA3-6B2, BD), anti-CD21-APC (7G6, BD), and anti-CD23-PE (B3B4, BD). For IgG-secreting plasma cells, after surface staining of anti-CD138-APC (IB17-R0268, Miltenyi), cells were fixed, permeabilized, and stained for anti-IgG-FITC (Poly4060, BD). For MZM staining, cells were stained with anti-F4/80-PE (BM8, Biolegend), anti-CD11b-Alexa Fluor 488 (M1/70, Biolegend), anti-SIGNR1-APC (22D1, eBioscience), and anti-MHC II (I-Ab)-eFluor 450 (AF6-120.1, eBioscience). All samples were detected on a flow cytometer (Beckman Coulter, USA) and analyzed with Cytexpert 2.0 software.

### Isolation and Culture of Bone Marrow-Derived Macrophages

To obtain bone marrow-derived macrophages (BMMs), C57BL/6, FcγRII^−/−^, and FcγRIII^−/−^ mice were sacrificed by cervical dislocation, and the bone marrow cells were flushed from the femoral shafts using serum-free DMEM. After the adherent cells were removed at 3 h, the non-adherent cells in suspension were cultured with 30 ng/ml M-CSF (PeproTech) for 6 days, and the medium was changed every 3 days. After incubation, the adherent BMMs were obtained. The adherent BMMs were then cultured in fresh complete culture medium for stimulation with 50, 100, 200, and 300 μg/ml lupus IgG that extracted from the SLE patient serum.

### Western Blotting

Isolated BMMs were lysed in RIPA buffer. The cell lysates were subjected to SDS-PAGE and transferred to polyvinylidene difluoride (PVDF) membranes (Millipore). The primary antibodies p-Syk (CST, 2717), Syk (CST, 2712), p-NF-κB p65 (CST, 3033), and NF-κB p65 (CST, 4764) were diluted with 1/1,000. The evaluation of immunoreactivity was performed with the ECL analysis system.

### ELISA Assay

TNF-α and MCP-1 levels were measured using ELISA kits (R&D Systems, USA). The blood serum and cell supernatant were diluted and studied using a standard curve. All of the samples were measured in triplicate. The procedure was performed according to the manufacturer's instructions.

Anti-dsDNA antibody levels in the serum were determined by ELISA, as described previously (32). Briefly, 96-well microtiter plates (Costar) were pretreated with calf thymus dsDNA (Sigma-Aldrich) for 2 h at 37°C and then placed overnight at 4°C. After being washed with PBS containing 0.05% Tween-20 (PBST), the plates were blocked with 1% BSA for 1 h. After the plates were incubated with a 1:100 dilution of mouse serum, the levels of anti-dsDNA antibodies were detected with horseradish peroxidase (HRP)-conjugated goat anti-mouse IgG, anti-mouse IgG1, anti-mouse IgG2a, anti-mouse IgG2b, anti-mouse IgG2c, anti-mouse IgG3, anti-mouse IgM, anti-mouse IgA, and anti-mouse IgE (all from Southern Biotech). Tetramethylbenzidine (TMB) substrate was used for the development, and absorbance at 450 nm was measured on a Thermo Multiskan Spectrum 1500.

### Statistical Analysis

All data are shown as the mean ± SEM. Statistical analyses were performed using GraphPad PRISM with Student's *t-*test. The differences were considered significant at ^*^P < 0.05, ^**^P < 0.01, and ^***^P < 0.001. All experiments were repeated at least three times with four to five mice in each group.

## Results

### Lupus Mice Spontaneously Develop Splenomegaly

**Figure 1:**
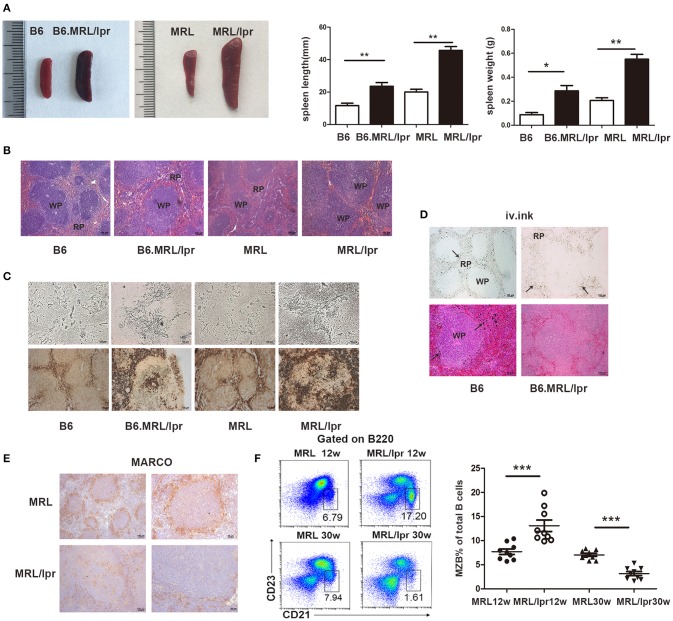
MRL/lpr mice spontaneously developed splenomegaly. **(A)** The size and weight of the spleens of 30-week-old B6.MRL/lpr, 26-week-old MRL/lpr mice and C57BL/6, MRL mice of the same age. **(B)** The representative histopathology of the spleens of 30-week-old B6.MRL/lpr, 26-week-old MRL/lpr mice, and C57BL/6, MRL mice of the same age. **(C)** Silver impregnation (upper part) and positive acid phosphatase reaction (under part) in the spleens of B6 (30 weeks), B6.MRL/lpr (30 weeks), MRL (26 weeks) and MRL/lpr mice (26 weeks). **(D)** Ink injection evaluation of the physiological function of the spleens of B6 mice (30 weeks) and B6.MRL/lpr mice (30 weeks). Carbon particles (arrow markers) distributed in the marginal zone of B6 mice and in the red pulp of B6.MRL/lpr mice. **(E)** MARCO expression in the spleens of MRL and MRL/lpr mice (30 weeks). **(F)** FACS analysis of the frequency of MZB cells (B220+CD21/35^hi^CD23^lo^) in the spleens of MRL/lpr (12 weeks and 30 weeks) and control MRL mice (12 weeks and 30 weeks). Data are representative of three experiments, *n* = 4–5 mice per group. WP, white pulp; RP, red pulp. *P < 0.05, **P < 0.01, ***P < 0.001.

To understand the changes in the spleen during SLE, we observed lupus mice that spontaneously develop lupus-like clinical manifestations. We found that splenomegaly spontaneously develops in B6.MRL/lpr and MRL/lpr mice. The length and weight of the lupus mice spleens were much greater than those of the normal mice (Figure 1A). Histopathology showed increased numbers of accumulated cells in the white pulp in the spleens of lupus mice (Figure 1B).

To investigate the architectural changes in the spleen in lupus mice, we used silver impregnation to assess the reticular fibers. We found increased reticular fiber density in the white pulp. Acid phosphatase staining revealed increased numbers of phagocytes in the splenic red pulp of the lupus mice (Figure 1C). We used ink injection to evaluate the physiological function of the spleen and found that carbon particles were disseminated into the red pulp of lupus mice, while particles were retained exclusively in the marginal zone of normal mice (Figure 1D). These data suggest that the antigen capture ability is weakened in the spleens of lupus mice. Since MZMs act as a barrier to the entry of circulating pathogens into the follicles of the spleen, we investigated the distribution of MZMs in the spleens of lupus MRL/lpr mice. We found that the numbers of MARCO+ cells were significantly reduced in the spleens of lupus MRL/lpr mice (Figure 1E). MZBs, which are an innate non-recirculating B cell population responsible for antigen transport from the marginal zone into the follicles and can be induced to differentiate rapidly into plasma cells, are located close to MZMs. We found that the MZB cell population was largely expanded in the marginal zone of the spleen at 12 weeks in MRL/lpr mice but decreased at 30 weeks in MRL/lpr mice (Figure 1F). These data suggest that there are structural defects in the spleen in lupus mice.

### IgG Production and Deposition Are Involved in the Pathogenesis of Splenomegaly in Lupus Mice

**Figure 2:**
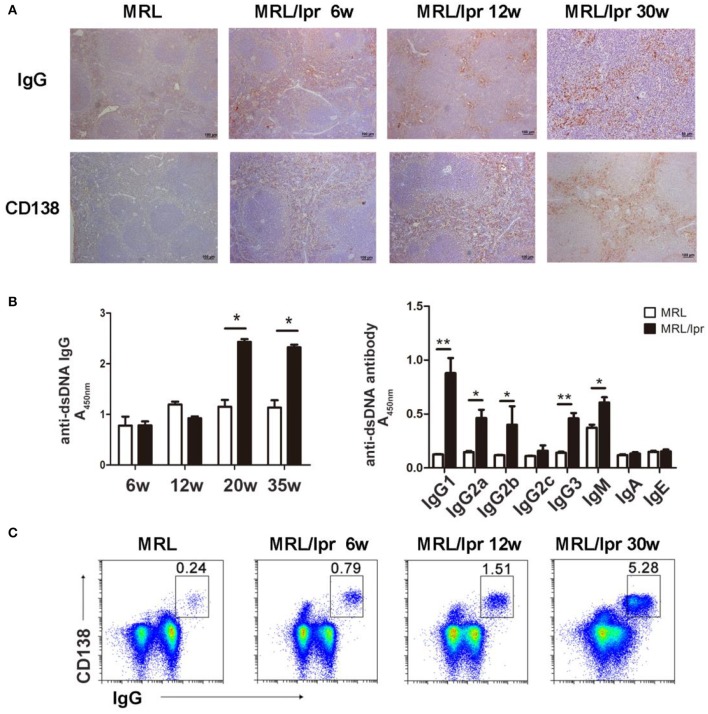
IgG production and deposition in the spleens of lupus mice. **(A)** IHC showing that IgG and CD138 are distributed in the splenic red pulp in 6-, 12-, and 30-week-old MRL/lpr mice. **(B)** Serum levels of the anti-dsDNA antibody in MRL/lpr mice (left panel) at different ages (6, 12, 20, and 35 weeks). Levels of anti-dsDNA antibody immunoglobulin isotypes in 35-week-old MRL/lpr mice (right panel). *P < 0.05, **P < 0.01. **(C)** Flow cytometry analysis of IgG-secreting plasma cells (IgG+CD138+) in 6-, 12-, and 30-week-old MRL/lpr mice. The results are representative of three independent experiments, *n* = 4–5 mice per group.

Since autoantibody-IgG is a major contributor to the development of tissue inflammation in SLE, we detected IgG deposition in the spleens of lupus MRL/lpr mice of different ages and found that IgG was deposited in the red pulp of the spleen in MRL/lpr mice of different ages. We also investigated plasma cells by detecting CD138 in the spleen and found that plasma cells were present in the red pulp in the spleens of mice of various ages (Figure 2A).

To determine whether the levels of autoantibodies in the peripheral blood reflect the levels of IgG deposition in the spleen of lupus mice, we detected immunoglobulin isotypes and anti-dsDNA antibodies. We found that the level of anti-dsDNA IgG in the blood increased as the age of MRL/lpr mice increased; IgG1, IgG2a, IgG2b, and IgG3 of anti-dsDNA were predominant in the blood of MRL/lpr mice (Figure 2B). Autoantibodies are generated by plasma cells; we found that the number of IgG-secreting plasma cells in the spleen increased from 6 to 30 weeks in MRL/lpr mice (Figure 2C). These results suggest that antibody-secreting cells produce pathogenic IgG autoantibodies that deposit in the spleen and secret into the periphery.

### Lupus IgG-induced Splenic Red Pulp Inflammation and GC Responses

**Figure 3:**
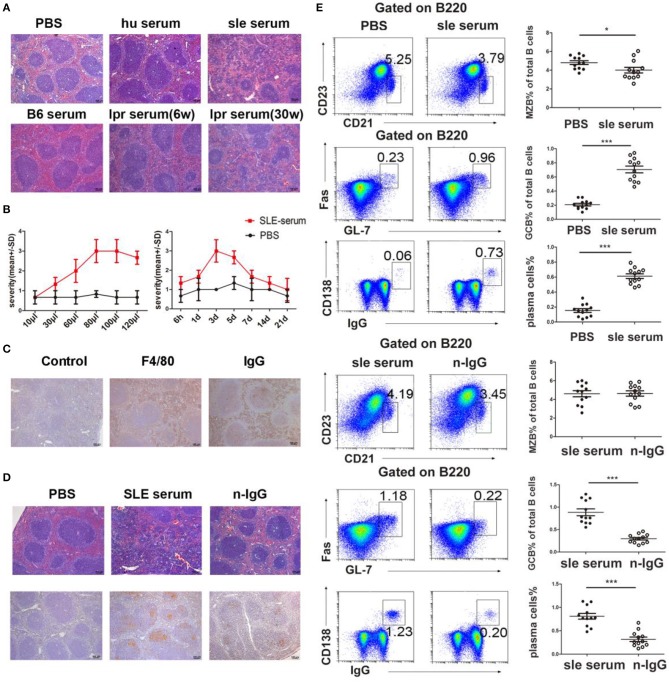
Lupus IgG-induced splenic red pulp inflammation and GC responses. **(A)** Histopathological image of the spleen of C57BL/6 mice at 3 days after intrasplenic injection of serum (80 μl) from healthy individuals, SLE patients, B6.MRL/lpr mice (30 and 6 weeks), and serum from C57BL/6 mice; PBS injection was used as a control. **(B)** Severity of red pulp inflammation induced by intrasplenic injection with different volumes of serum from a lupus patient (left panel), and the kinetics of inflammation on different days after intrasplenic injection of lupus serum (80 μl) (right panel). **(C)** IHC results of F4/80 and IgG staining in the spleen of C57BL/6 mice 3 days after the injection of SLE patient serum. **(D)** Histopathological image of red pulp inflammation 3 days after injection of PBS, SLE patient serum, and IgG-depleted SLE patient serum. IHC results of peanut agglutinin (PNA) 8 days after injection of PBS, SLE patient serum, and IgG-depleted SLE patient serum. PNA staining was used for GC identification. **(E)** FACS analysis of the frequency of MZB cells (B220+CD21/35^hi^CD23^lo^), germinal center B (GCB) cells (B220^+^Fas^+^GL7^+^), and IgG-secreting plasma cells (IgG^+^CD138^+^) 8 days after intrasplenic injection of PBS, SLE patient serum, SLE patient serum, and IgG-depleted SLE patient serum in the spleens of C57BL/6 mice. All results are representative of three independent experiments; *n* = 4–5 mice per group. *P < 0.05, ***P < 0.001.

In this study, we found a large amount of IgG secretion and deposition in extrafollicular foci in the spleens of the lupus mice (Figure 2). To confirm that deposited IgG further induces immune responses in the spleen, we established a model with IgG deposition in the mouse spleen by intrasplenic injection of serum from SLE patients and healthy individuals. The results showed that serum from SLE patients induced red pulp inflammation, while PBS and serum from healthy humans did not (Figure 3A). We further used serum from lupus mice and normal mice to repeat this experiment. We also found that serum from lupus mice induced inflammation in the spleen, but serum from normal mice and young lupus mice did not (Figure 3A). To determine whether the severity of inflammation induced by lupus serum in the spleen is related to the volume injected, we used various volumes of lupus serum. We found that the severity of inflammation in the spleen is related to the volume of injected lupus serum (Figure 3B). We also investigated the kinetics of inflammation induced by lupus serum in the spleen and found that inflammation developed at 6h and peaked at 3 days after intrasplenic injection (Figure 3B). We used IHC staining to determine the cell types in the inflamed spleens. We found that spleen inflammation was characterized by the infiltration of F4/80 macrophages in the red pulp (Figure 3C). IgG deposition was observed in the spleens of C57BL/6 mice receiving intrasplenic injections of lupus serum (Figure 3C). We did not observe any inflammation and IgG deposition following intravenous and intraperitoneal injection of the same volume of lupus patient serum (Figure S1).

To confirm the role of IgG in inflammation induced by lupus serum, we used IgG-depleted lupus serum. We found that at 3 days after injection, the severity of inflammation was significantly decreased in the red pulp of mice injected with IgG-depleted SLE serum compared to mice injected with whole SLE serum (Figure 3D). Lupus IgG from SLE serum significantly induced spleen inflammation compared to IgG from healthy human serum (Figure S2). This finding suggests that IgG plays an important role in the development of inflammation induced by lupus serum in the spleen. MZB cells located in the marginal zone of the spleen differentiate directly into plasma cells in extrafollicular responses. GCs are important sites of autoreactive B cell activation in autoimmune diseases. Antibodies are secreted by plasma cells in the spleen through extrafollicular and GC pathways (11). We investigated whether lupus serum IgG induces extrafollicular responses, GC responses, and plasma cell formation using flow cytometry. The results showed that the numbers of GC and IgG-secreting plasma cells increased at 8 days after injection of SLE patient serum, whereas GC and plasma cell numbers decreased in mice injected with IgG-depleted SLE serum (Figure 3E). We found an approximately five-fold reduction in GCs and plasma cells in the spleens of mice injected with IgG-depleted SLE serum compared to mice injected with whole SLE serum. MZB cell numbers decreased at 8 days after injection of SLE patient serum. There were no significant changes in MZB cell numbers between mice injected with SLE patient serum and mice injected with IgG-depleted SLE patient serum. The injection of healthy human serum did not induce obvious changes in GCs and plasma cell numbers (Figure S3). These data suggest that IgG is an important factor in the development of lupus serum-induced red pulp inflammation and the GC response.

### Macrophages Are Required for the Development of Spleen Inflammation and GC Responses Induced by Lupus IgG

It has been reported that macrophages play a critical role in tissue damage in SLE (18). It is unclear whether macrophages are involved in spleen inflammation and the GC response induced by lupus IgG.

**Figure 4:**
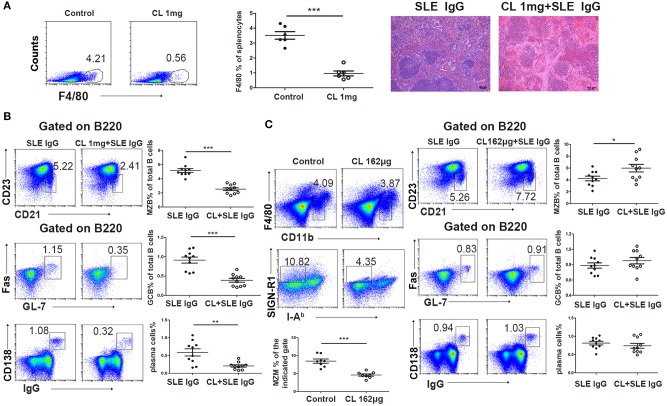
Effect of macrophages on lupus IgG-induced red pulp inflammation and GC responses. **(A)** FACS analysis showing that F4/80 expression in the spleen of C57BL/6 mice depleted of red pulp macrophages with 1 mg of CLs is significantly decreased compared with that in control mice treated with PBS liposomes. Representative histopathological picture of the spleen of C57BL/6 mice 3 days after intrasplenic injection of lupus IgG (200 μg), and lupus IgG (200 μg) pretreated with 1 mg of CLs. **(B)** FACS analysis of MZB (B220+CD21/35^hi^CD23^lo^), GCB (B220^+^Fas^+^GL7^+^), and IgG-secreting plasma cells (IgG^+^CD138^+^) 8 days after intrasplenic injection of lupus IgG (200 μg), and lupus IgG (200 μg) pretreated with 1 mg of CLs. **(C)** FACS analysis of the percentage of MZMs (F4/80^neg^CD11b^lo^SIGN-R1^+^I-A^b−^) in the spleen of C57BL/6 mice injected with 162 μg of CLs and PBS liposomes as the control. Frequency of MZB (B220^+^CD21/35^hi^CD23^lo^), GCB (B220^+^Fas^+^GL7^+^), and IgG-secreting plasma cells (IgG^+^CD138^+^) 8 days after intrasplenic injection of lupus IgG (200 μg), and lupus IgG (200 μg) pretreated with 162 μg of CLs. All results are representative of three independent experiments, *n* > 10. *P < 0.05, **P < 0.01, ***P < 0.001.

To determine the role of macrophages in lupus IgG-induced spleen inflammation and the GC response, we depleted macrophages in experimental mice by using various doses of CLs (33). We treated mice with high (40 mg/kg) and low doses (6.5 mg/kg) of CLs, which depleted red pulp macrophages, and MZMs, respectively. We found that the inflammation induced by lupus IgG was alleviated in red pulp macrophage-depleted mice compared to mice without depletion of red pulp macrophages (Figure 4A). This finding suggests that red pulp macrophages are crucial for the initial inflammation induced by lupus IgG. Since MZMs expressing MARCO are deficient in the spleens of lupus MRL/lpr mice (Figure 1E), we further determined whether lupus IgG affects GC and plasma cells in red pulp macrophages and the MZM deficiency. The results showed that depletion of red pulp macrophages (pretreated with 40 mg/kg CLs) decreased MZB, GCB, and plasma cell populations in the spleens of mice injected with lupus IgG (Figure 4B). MZM deficiency (pretreatment with 6.5 mg/kg CLs) increased the number of MZB cells, which may respond to lupus IgG, but did not affect GCB and plasma cell populations (Figure 4C; Figure S4). These data suggest that lupus IgG can promote the formation of GCs and plasma cells without MZMs. Red pulp macrophages are indispensable for the development of spleen inflammation and the formation of GCB and plasma cells.

### The Role of Syk, FcγR, and TNF-α in Inflammation and the GC Response Induced by Lupus IgG

**Figure 5:**
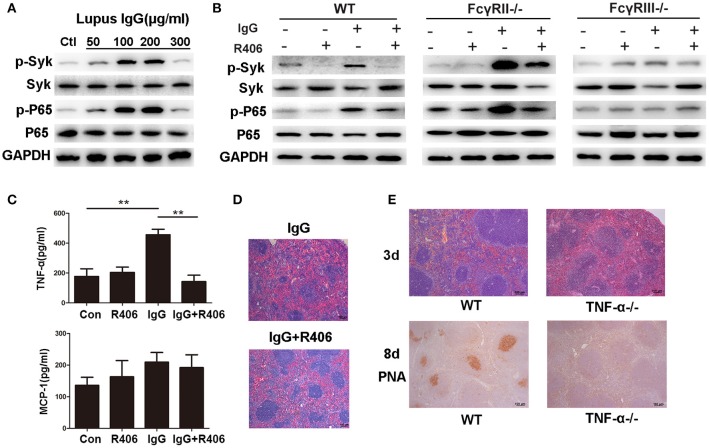
Lupus IgG promoted inflammation and GC formation through TNF-α secretion by macrophages. **(A)** Western blot detected phosphorylated Syk (p-Syk) and total Syk, phosphorylated NF-κB p65 (p-p65), and p65 in BMMs stimulated with various doses of lupus IgG for 20 min. The results are from three independent experiments. **(B)** Western blot detected p-Syk, Syk, p-p65, and p65 in BMMs stimulated with 100 μg/ml lupus IgG in the presence or absence of R406 (2 μM) for 30 min. BMMs were isolated from WT, FcγRII^−/−^, and FcγRIII^−/−^ mice. The results are from three independent experiments. **(C)** ELISA results of TNF-α and MCP-1 in the supernatant of WT-derived BMMs stimulated with 100 μg/ml lupus IgG for 18 h in the presence or absence of R406 (2 μM). **P < 0.01, the result is representative of three independent experiments. **(D)** Histopathological image of the spleen in C57BL/6 mice treated with or without the Syk inhibitor R406 (10 mg/kg) after 3 days of intrasplenic injection of lupus IgG (200 μg) from an SLE patient. The results are representative of three independent experiments, *n* = 5 mice per group. **(E)** Histopathological image of the spleen 3 days after intrasplenic injection of lupus IgG (200 μg) in WT (C57BL/6) and TNF-α^−/−^mice. PNA was expressed 8 days after intrasplenic injection of lupus IgG in WT and TNF-α^−/−^ mice. The result is representative of three independent experiments, *n* = 5 mice per group.

Syk plays an important role in lupus IgG-induced signal transduction. We examined the lupus IgG-induced Syk-NF-κB signaling pathway in BMMs and found that lupus IgG induced Syk and NF-κB p65 phosphorylation in a dose-dependent manner (Figure 5A; Figure S5). To further confirm the effect of lupus IgG on the activation of Syk and NF-κB, we used a Syk inhibitor. We found that the Syk inhibitor treatment suppressed p-Syk and NF-κB p-p65 activation induced by lupus IgG (Figure 5B).

The FcγRs, which are IgG receptors, include activating receptors FcγRI and FcγRIII and the inhibitory receptor FcγRII. We used FcγRII- and FcγRIII-deficient BMMs to determine the role of these receptors in the lupus IgG-induced Syk-NF-κB signaling pathway. The results showed that deficiency of the activating receptor FcγRIII decreased Syk and NF-κB p65 activation induced by lupus IgG, while deficiency of the inhibitory receptor FcγRII increased Syk and NF-κB p65 activation induced by lupus IgG (Figure 5B).

To investigate the cytokine release by macrophages stimulated with lupus IgG, we detected TNF-α and MCP-1 levels in the supernatant of BMMs stimulated with lupus IgG. We found that lupus IgG increased the levels of TNF-α and MCP-1, whereas Syk inhibitor treatment significantly inhibited TNF-α and decreased MCP-1 expression, but the difference in MCP-1 expression was not significant (Figure 5C).

To further understand whether Syk regulates spleen inflammation induced by lupus IgG, we compared changes in the histopathology of spleens from mice treated with or without a Syk inhibitor. The results showed that the Syk inhibitor treatment obviously decreased the red pulp inflammation induced by lupus IgG (Figure 5D). Since lupus IgG increased the level of TNF-α in the supernatant of BMMs, we speculated that lupus IgG could promote GC formation through the secretion of TNF-α by macrophages. To confirm this speculation, we used TNF-α-deficient mice. The histopathology demonstrated that red pulp inflammation induced by lupus IgG was significantly decreased in TNF-α-deficient mice compared to wild-type (WT) mice. The IHC staining showed that GC formation induced by lupus IgG was also significantly reduced in TNF-α-deficient mice compared to WT mice (Figure 5E). In TNF-α-deficient mice, we found that lupus IgG decreased the initial red pulp inflammation and further inhibited GC formation.

## Discussion

Splenomegaly has been reported in lupus mice and patients, but there is little information available about the mechanism of splenomegaly in SLE patients. Although the pathogenesis of SLE remains unclear, evidence suggests that SLE may be caused by immunological abnormalities, including immunotolerance deficiency, abnormal interactions of T and B cells, immune cell hyperactivity, and defective clearance of autoantigens (34). The spleen is the largest peripheral lymphoid organ and has a specialized structural organization for immunocyte proliferation and differentiation, and interaction with the circulation. Sustained autoantigen and immune complex deposition in the spleen may enhance the abnormalities and promote the progression of SLE. Our study demonstrates that lupus mice spontaneously develop splenomegaly, and the architectural injury of the spleen in lupus mice may be due to the production and deposition of large amounts of pathogenic IgG, which is an important pathological factor in spleen inflammation and immunological abnormalities.

SLE is a disease mainly driven by autoantibodies (35). We found large amounts of IgG and quantities of plasma cells in the spleens of MRL/lpr mice; the accumulation of anti-dsDNA IgG in the peripheral blood is related to age. The dsDNA antibody is an important parameter for both diagnosis and treatment in SLE (36, 37). It has been reported that autoantibodies in SLE are mainly produced by B cells through affinity maturation and somatic hypermutation in GCs and also extrafollicular responses (11, 38). Plasma cells can arise through B cell activation and direct differentiation in the extrafollicular site or in the T-dependent GC response. Antigens can activate B cells in a T-independent or T-dependent manner. There are no definite markers that discriminate plasma cells by their pathway of differentiation. We and others have shown that IgG isolated from SLE patient serum can induce organ damage (18, 20, 39). Therefore, we established animal models of IgG deposition in the spleen by intrasplenic injection of lupus serum. Based on this model, we investigated whether lupus IgG induced inflammation and further GC and plasma cell formation. The results showed that the intrasplenic injection of lupus serum-induced red pulp inflammation at 3 days and further increased GC and plasma cells differentiation at 8 days. However, compared to mice injected with lupus serum containing IgG, mice injected with IgG-depleted lupus serum displayed decreased GC and plasma cell formation. Therefore, lupus IgG in SLE serum is the important pathological factor inducing red pulp inflammation, and dysfunctional adaptive immune responses in the spleen in lupus.

It has been shown that MZMs surrounding the splenic follicles can clear autoantigens or blood-borne antigens. The absence of MZMs results in the retention of apoptotic cell debris within the marginal zone and increased loading of antigens on MZB cells, stimulating the autoimmune response (3). MZMs have been reported to interact with MZB cells in the spleens of BXD2 lupus mice with the membrane lymphotoxin-α1β2 and lymphotoxin β receptor. The abnormal follicular shuttling of MZB cells causes the loss of MZMs and thus downregulates the clearance of autoantigens (40). Our data demonstrate that depletion of MZMs increased the number of MZB cells, but did not affect the presentation of antigens and induction of the GC response, which is consistent with a report by Aichele (41). MZB cells can differentiate directly into plasma cells in extrafollicular responses and can also deliver antigens to the follicles, enhancing T-dependent responses (42). We found that lupus serum IgG decreased MZB cell numbers, but it is not clear if lupus IgG affects extrafollicular responses or the delivery of antigens to the follicles. MZMs and red pulp macrophages are known to interact with apoptotic cells entering the spleen from the circulation. There are many studies on the defects in the MZM-mediated regulation of inflammation and tolerance in autoimmune diseases, but not many on red pulp macrophages. Red pulp macrophages are known to increase the phagocytosis of apoptotic cells in the absence of MZMs (33), but information on how red pulp macrophages regulate inflammation and adaptive immunity in autoimmune diseases is lacking. Macrophages in the red pulp of the spleen express receptors for IgG or IgG-opsonized erythrocytes (43). In our study, red pulp macrophages were found to influence inflammation and GC responses induced by lupus IgG. The removal of F4/80 splenic macrophages can inhibit the development of autoantibodies and lupus pathology in mice (33). Therefore, this finding suggests that macrophages play a role in the pathogenesis of SLE.

IgG deposition in tissue can activate complement or engage in the activation of the FcγR to mediate antibody-dependent cell cytotoxicity (ADCC) and initiate inflammation (44). In this study, we found that the effect of lupus IgG was mediated by its binding to the FcγR on macrophages. Lupus IgG activated Syk and NF-κB signaling, leading to the release of TNF-α. In the *in vivo* experiment, we found that red pulp macrophages play an important role in initial inflammation and GC and plasma cell formation. Lupus IgG promotes inflammation though macrophage-mediated secretion of TNF-α and further promotes GC formation. TNF-α is important for the development of skin and liver injuries induced by lupus serum (18, 20). However, whether macrophages interact with T cells induced by lupus IgG requires further investigation. Syk plays a central role in FcγR-mediated macrophage activation. In the current study, we inhibited Syk downstream of FcγR, which decreased the inflammation induced by lupus IgG, and decreased NF-κB signaling and TNF-α secretion induced by lupus IgG in macrophages. Numerous studies have described Syk abnormalities in BCR-mediated signaling in B cells and Syk inhibition in the progression of glomerulonephritis in SLE (45), but there is little information regarding the role of FcγR-mediated signaling in the progression of spleen inflammation and immune responses. Syk is an important therapeutic target for the spleen inflammation. FcγR-mediated signaling transduction is dependent on immune-receptor tyrosine-based activation motifs (ITAM), which are located in its cytoplasmic tail. Syk binds these phosphorylated ITAM motifs and activates downstream signaling. We confirmed that inhibiting Syk suppressed spleen inflammation and macrophage activation in the vitro experiment and spleen inflammation model. Furthermore, we observed that inhibitor of Syk also treated splenomegaly in our previous study (29).

In conclusion, our study demonstrates that IgG plays an important role in the pathogenesis of splenomegaly in lupus mice. IgG production and deposition in the spleen is an important pathological factor in spleen inflammation and immunological abnormalities. Red pulp macrophages, instead of marginal zone macrophages, are indispensable for inflammation and GC responses in splenomegaly. Lupus IgG promotes the GC response through TNF-α production by macrophages. Syk is a therapeutic target for the suppression of inflammation in splenomegaly in SLE. These findings will contribute to future research for the development of therapeutic strategies for splenomegaly in SLE.

## Data Availability Statement

All datasets generated for this study are included in the article/Supplementary Material.

## Ethics Statement

This study was carried out in accordance with the recommendations of the Institutional Animal Care and Use Committee (IACUC) guidelines of Nanjing Medical University. The protocol was approved by the Institutional Ethics Committee of Nanjing Medical University.

## Author Contributions

QZ and G-MD designed the research and wrote the manuscript with the contribution from MZ. QZ, LX, and WD performed the experiments. QZ and GH analyzed the data for this manuscript.

### Conflict of Interest

The authors declare that the research was conducted in the absence of any commercial or financial relationships that could be construed as a potential conflict of interest.
