# Supplementary material for: Predominant Role of Immunoglobulin G in the Pathogenesis of Splenomegaly in Murine Lupus
Source: Front Immunol. 2020 Jan 24;10:3020. doi: 10.3389/fimmu.2019.03020 (PMC7005523; doi:10.3389/fimmu.2019.03020)
Supplement: Supplementary file 1 [file Image_1.pdf]

Supplementary Material

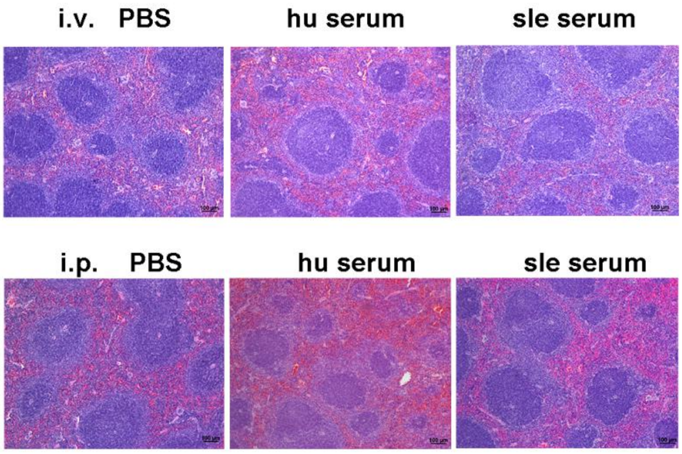

Figure S1. The histological structure of the spleen 3 days after intravenous injection (i.v.) and intraperitoneal injection (i.p.) of PBS, healthy human serum, and SLE patient serum (80 µl). The results are representative of three independent experiments, n = 5 mice per group. HE staining.

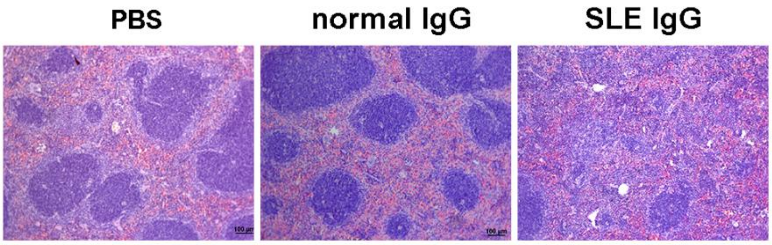

Figure S2. The histological structure of the spleen 3 days after intrasplenic injection of PBS, normal IgG (200 µg) from healthy human serum, SLE IgG (200 µg) from SLE serum. The results are representative of three independent experiments, n = 5 mice per group. HE staining.

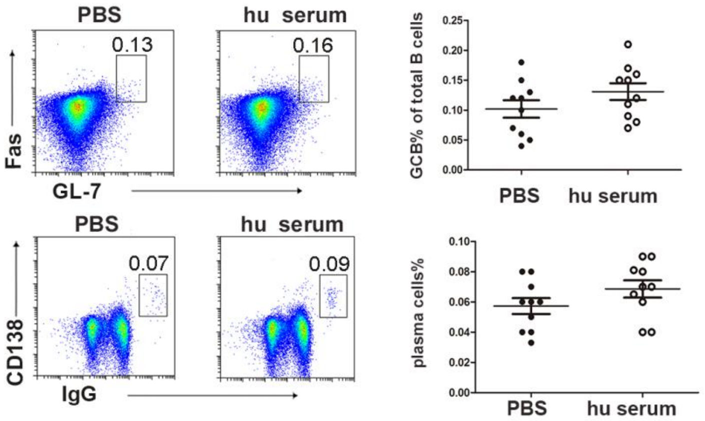

Figure S3. Flow cytometry analysis of GCB (B220+Fas+GL7+) and IgG-secreting plasma cells (IgG+CD138+) 8 days after intrasplenic injection of PBS or healthy human serum. All results are representative of three independent experiments, n = 4-5 mice per group.

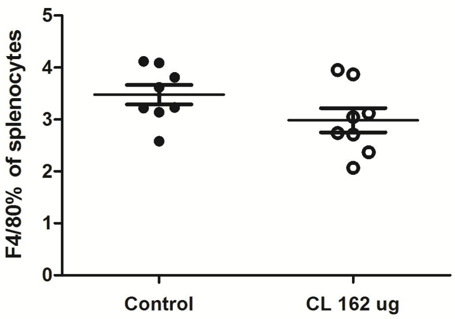

Figure S4. FACS analysis of the percentage of F4/80 macrophages in the spleen of C57BL/6 mice injected with 162 μg of CLs and PBS liposomes as the control. There was no significant effect on F4/80 macrophages after injection of 162 μg CLs.

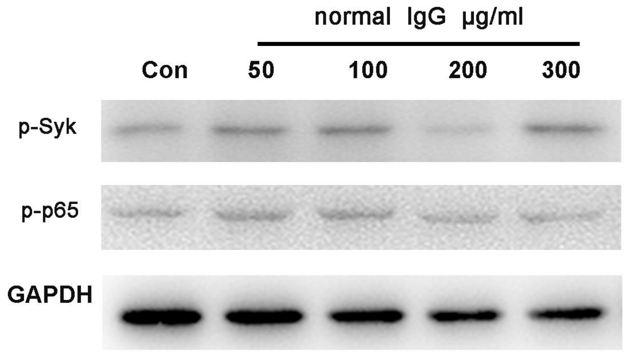

Figure S5. Western blot detected phosphorylated Syk (p-Syk) and phosphorylated NF-κB p65 (p-p65) in BMMs stimulated with various doses of normal human IgG for 20 min. The results are from three independent experiments.
